# A Survey of Home Enteral Nutrition Practices and Reimbursement in the Asia Pacific Region

**DOI:** 10.3390/nu10020214

**Published:** 2018-02-14

**Authors:** Alvin Wong, Merrilyn D. Banks, Judith D. Bauer

**Affiliations:** 1 Dietetic and Food Services, Changi General Hospital, Singapore 529889, Singapore; 2 School of Human Movement and Nutrition Sciences, University of Queensland, St Lucia QLD 4072, Queensland, Australia; j.bauer1@uq.edu.au; 3 Department of Nutrition and Dietetics, Royal Brisbane and Women’s Hospital, Herston QLD 4029, Queensland, Australia; Merrilyn.Banks@health.qld.gov.au

**Keywords:** home enteral nutrition, nutrition support team, reimbursement, funding, nutrition education

## Abstract

Literature regarding the use of home enteral nutrition (HEN) and how it is reimbursed in the Asia Pacific region is limited. This research survey aims to determine the availability of HEN, the type of feeds and enteral access used, national reimbursement policies, the presence of nutrition support teams (NSTs), and clinical nutrition education in this region. An electronic questionnaire was sent to 20 clinical nutrition societies and leaders in the Asia Pacific region in August 2017, where thirteen countries responded. Comparison of HEN reimbursement and practice between countries of different income groups based on the World Bank’s data was investigated. Financial support for HEN is only available in 40% of the countries. An association was found between availability of financial support for HEN and health expenditure (*r* = 0.63, *p* = 0.021). High and middle-upper income countries use mainly commercial supplements for HEN, while lower-middle income countries use mainly blenderized diet. The presence of NSTs is limited, and only present mainly in acute settings. Sixty percent of the countries indicated an urgent need for funding and reimbursement of HEN. This survey demonstrates the varied clinical and economic situation in the Asia Pacific region. There is a lack of reimbursement, clinical support, and inadequate educational opportunities, especially for the lower-middle income countries.

## 1. Introduction

Home enteral nutrition (HEN) is a life-saving and life-sustaining therapy for patients who are unable to obtain adequate nutrition via the oral route. The main indications for HEN include swallowing disorders, the need to improve nutritional status of patients, and gastrointestinal problems such as obstruction and malabsorption [1]. The yearly prevalence of HEN was estimated at 463 per million population in the United States [2], with an incidence of 163–360 per million population in the United States and Europe in the 1990s [2,3]. It was also estimated that about 2–34% of residents in nursing homes in the United States were on HEN [4]. In the United Kingdom, the latest British Artificial Nutrition Survey (BANS) reported a 5% increment in HEN between 2009 and 2010, with an incidence of 55 per million population and point prevalence of 92 per million population [1].

An early study in the United States reported the cost of home enteral nutrition, including feeds, supplies, and care, and one hospitalization stay to range from USD $5000 to $50,000 [5]. This price is likely to have increased in recent years, although it is generally difficult to obtain expenditure information now given differences in insurance coverage and reimbursement. Reimbursement for enteral nutrition has been made available to patients in the United States and some European nations for many years. The availability and proportion of reimbursement, however, varies greatly between countries and even within states/areas of individual countries.

### 1.1. HEN Reimbursement in North America

In the United States, third party payers such as the Centers for Medicare and Medicaid Services provide funding for HEN patients [6]. The American reimbursement system is complicated and a strict criteria is needed for reimbursement, which includes the presence of a disease state that impairs the ability of the gut for >90 days; inability to obtain sufficient nutrition through diet or oral nutrition supplementation nutrition; supplementation must be via a feeding tube; and the requirement of medical documentation [7,8]. Medicare pays 80% or less of the actual charge for the specific item, with the remaining 20% being the responsibility of the beneficiary or a secondary payer [7]. In Canada, reimbursement of HEN varies between the provinces and territories. Funding appears to be from governmental agencies, such as the Provincial Health Services Authority for British Columbia and Ministry of Health and Long-Term Care for Ontario. In Ontario, long-term care homes are reimbursed an additional Canadian CAD$0.12 per resident per day [9]. There is also other funding available from insurance and for veterans [10].

### 1.2. HEN Reimbursement in Europe

HEN reimbursement and availability varies greatly in European nations. According to a recent survey done by Klek et al. [11], HEN is not reimbursed in low-middle income countries in Europe such as Ukraine, but is reimbursed in upper-middle income countries (Serbia and Turkey) and high-income countries (Croatia, Czech Republic, Estonia, Finland, Germany, Greece, Ireland, Italy, Latvia, Netherlands, Norway, Poland, Russia, Spain, Switzerland, and the United Kingdom). Costs of HEN are fully funded by private or national health insurance in France, Germany, Italy, Spain, Switzerland, and the UK [12,13,14]. There is partial reimbursement of 60% in Denmark, but a 3-month medical prescription is required [15]. In Belgium, only 30% of HEN is funded by the National Health Service [13].

### 1.3. HEN Reimbursement in the Asia Pacific Region

In the Asia Pacific region, the majority of the literature available on HEN reimbursement and funding is from Australia. The direct overhead costs associated with the provision of services (e.g., enteral feeds, consumables, blood tests, manpower) in a recent report of 1329 HEN patients was approximately AU$1.53 million, of which AU$1.09 million (AU$817/patient) was paid by the hospitals [16]. The remainder was paid via co-payments or full patient contributions. Variable practices occur across states/territories as well as between hospitals within the same jurisdiction in Australia. HEN is fully funded only in the Northern Territory, Australian Capital Territory, South Australia, Tasmania, and Victoria, but the sources of funding vary. Other states require patient co-payments for consumables and medical surgical supplies, or require patients to cover the costs associated with feeds [16].

There is limited literature published in the English language on nutrition reimbursement in the other Asia Pacific countries. A Japanese study reported that patients with irreversible cognitive impairment who require long-term HEN are often cared for in long-term care hospitals [17]. In another study by Suzuki et al. [18] of 202 hospitals in Japan, only 14.3% of patients with a percutaneous endoscopic gastrostomy (PEG) inserted in the hospitals were discharged home with a PEG. The majority of these patients were admitted to long-term care hospitals. For patients in these long-term care hospitals, the inclusive per diem payment system covers for HEN [17,18]. It is noted that HEN is covered by the Japanese National Health Insurance for certain diseases such as Crohn’s disease [19] and claimable from insurance for most of the other patients [18]. In South Korea, only publications on reimbursement for inpatient nutrition support team (NST) activities are available [20,21], although Klek et al. [22] reported the availability of reimbursement in South Korea recently. Most of the countries in Southeast Asia do not reimburse HEN or enteral nutrition in acute settings. For example, the cost of home enteral feeding is not funded in Malaysia, with an average cost of enteral feeds estimated at RM 830 per month [23]. 

The latest international surveys on nutritional funding and reimbursement were performed by Klek et al. [11,22] in 2014 and 2015. However, the surveys did not include some of the countries in Asia and given the dearth of literature available in this region, an updated regional survey is warranted. The aims of this research survey are to determine the (a) presence of enteral nutrition usage in acute and chronic care settings; (b) type of enteral feeds used (commercial versus blenderized diets); (c) types of enteral access; (d) national/state reimbursement policies; (e) presence of nutrition support teams; and (f) availability of clinical nutrition training in the Asia Pacific region.

## 2. Materials and Methods

A modified version of the survey questionnaire (Supplementary Material Figure S1) by Klek et al. [11] was sent electronically to the Parenteral and Enteral Nutrition (PEN) societies and leaders of nutritional support in the Asia Pacific region in August 2017 over a period of three months. These countries include Australia, Brunei, Cambodia, China, Hong Kong, India, Indonesia, Iran, Japan, South Korea, Laos, Macau, Malaysia, Myanmar, New Zealand, Taiwan, the Philippines, Singapore, Sri Lanka, and Vietnam. The survey questions were categorized to the following sections: (A) Demographics; (B) Reimbursement for Enteral Nutrition; (C) Current Nutritional Practice; and (D) Training and Education for Clinical Nutrition in the Country. The term “home” used in the questionnaire referred to patients who were staying at home with their family/caregiver or alone, and the term “long-term care settings” included patients who were staying in nursing homes, retirement homes, step-down care hospitals, chronic care hospitals, and palliative homes/hospices. HEN applies to both enteral feeding at home and in long-term care settings. We also collected data on reimbursement for enteral nutrition in acute care settings (hospitals) for the purpose of comparison with HEN.

We invited a validation panel consisting of five experts in the field to determine the face and content validity of the modified survey. Validation panelists were tasked to review the survey for face and content validity via an online questionnaire, which incorporated a structured reporting form developed by the researcher. The validation form contained five sections—(A) Clarity; (B) Usefulness; (C) Preferred layout; (D) Content validity; and (E) Completion time. The questions in the survey were based on the questionnaire evaluation criteria described by Neuman [24], Berdie et al. [25], and Dillman [26].

The validation panelists were also requested to comment on the (A) clarity of words used in the questions/answers; (B) appropriateness of the questions and response categories; (C) comprehensiveness of the questions and response categories; (D) sequencing of the questions; (E) overall design of the questionnaire; and (F) how long it took to the panelists to complete the survey being validated, as we aimed to have a completion time of ≤20 min to minimize respondent fatigue [24]. The survey was revised based on the comments received from the validation panel.

Countries were categorized by their gross domestic product (GDP, purchasing power parity (PPP)) and gross domestic product (GDP, US dollars), income groups (low-income: ≤US$1005; low-middle income: US$1006–US$3955; upper-middle income: US$3956–12,235; high income: ≥US$12,236), as well as the health expenditure per capita based on data from the World Bank [27]. For special administrative regions such as Hong Kong and Macau whose data was not available from the World Bank database, information was obtained from the individual country’s health ministry website.

We performed statistical analysis using SPSS version 25 (IBM Corp., Armonk, NY, USA) software package. Values were reported as median with interquartile range (IQR). The Cochran-Armitage test of trend and point-biserial correlation were used to determine the relationships between variables. Mann-Whitney U test was used to determine differences between groups. A *p*-value of <0.05 was considered as statistically significant.

This study has been cleared in accordance with the ethical review guidelines at The University of Queensland, Australia (approval number HMS17/21). The study adheres to the Guidelines of the ethical review process of The University of Queensland and the National Statement on Ethical Conduct in Human Research.

## 3. Results

### 3.1. Demographics

Thirteen of twenty-one countries responded to the survey, a 62% response rate. The countries that participated were Australia, Brunei, Cambodia, Hong Kong, India, Indonesia, Japan, the Republic of Korea, Malaysia, Myanmar, New Zealand, the Philippines, and Singapore. All questions of the survey were completed by these 13 participating countries. Participants of the survey included dietitians and medical doctors who are experienced in HEN practice, or members of the respective PEN societies.

The demographics of the responding countries are as presented in Table 1. Seven countries are in the high-income, one in upper-middle income, and five in lower-middle income tiers. GDP (PPP) ranged from $32,773 to $5,266,444 (median: $806,539, IQR 2,187,786). GDP (US$) range from US$11,400 to US$4,939,384 (median: US$304,905, IQR: US$1,181,708). Health expenditure (public) as a percentage of GDP ranged from 1.10% to 9.10% (median: 2.30%, IQR: 4.65%). Healthcare regulation is developed by the state/governmental bodies in all the countries. They are also responsible for the development of nutritional guidelines in approximately 70% of the countries surveyed, with the remainder by various clinical societies. The only exception is Cambodia, which has no official nutritional guidelines.

### 3.2. Reimbursement

Approximately 75% of the countries have both state and private health insurance, and the remainder had either state or private insurance only, as shown in Table 2. Financial support for HEN was only available in 40% of the countries, and policies or guidance for financial support varied between states in approximately half of these countries. All the countries without HEN reimbursement reported either no plans (25%) or uncertain of plans (75%) for future governmental funding for HEN. Out-of-pocket payments by patients or families are required for HEN in these countries without reimbursement.

### 3.3. HEN: Home Enteral Nutrition

In countries with financial support for enteral nutrition in acute or home settings (Table 3), 83% have only partial funding, and the distribution of financial support is similar across all facilities (acute, long-term, palliative, and home care). The only exception is Singapore, which only provides partial financial support for enteral nutrition in the acute setting. The majority of the funding comes from state insurance, and private insurance reimbursement of HEN is only available in approximately 30% of these countries. There were no statistically significant correlations between availability of financial support for HEN and GDP (PPP) (*r* = 0.343, *p* = 0.252), HEN and GDP (US$) (*p* = 0.102), or HEN and income group (*r* = 0.474, *p* = 0.180). However, a statistically significant correlation was found between availability of financial support for HEN and health expenditure as a percentage of GDP (*r* = 0.63, *p* = 0.021).

### 3.4. Current Nutritional Practice

All the high and middle-upper income countries use mainly ready-to-use commercial supplements for HEN in home and long-term care settings. Lower-middle income countries use either blenderized diet exclusively (40%) or an equal amount of blenderized diet and commercial supplements (60%) in home settings, and a similar proportion (60% blenderized diets and 40% use of both) in long-term settings (Supplementary Material Table S1).

As described in Table 4, prescription of the nutritional care plan was carried out by dietitians and/or doctors in all the high and middle-upper income countries (*n* = 8), and mainly by doctors in middle-lower income countries (*n* = 5). Training of patients and caregivers on HEN administration is mainly carried out by nursing staff in most of the countries. Of note, there are no dietitians working in acute or long-term care settings in Myanmar and Cambodia. The presence of NST is also limited in most of the countries surveyed, and only present mainly in acute settings. There are no available or published statistics on HEN incidence or prevalence in all the countries surveyed.

### 3.5. Training and Education for Clinical Nutrition

As shown in Table 5, undergraduate clinical nutrition training (excluding dietetics course) is only available in less than 25% of the countries. There is no clinical nutrition training available in Brunei and Cambodia. Post-graduate, ESPEN (The European Society for Clinical Nutrition and Metabolism) Life Long Learning-, Dietetic Association-, and hospital-organized courses are available in more than 60% of the countries surveyed. There is no statistically significant difference (*U* = 28.5, *z* = 1.258, and *p* = 0.208) between income groups (high and middle-upper income versus lower-middle income) and availability of clinical nutrition training (total number of types of clinical training courses).

### 3.6. Other Issues

Sixty percent of the countries surveyed indicated an urgent need for funding and reimbursement of HEN from government/state or private insurance coverage. Seventy percent stressed on the need for adequate training in HEN support for all stakeholders (healthcare workers, patients, families, and the various organizations involved). Other important issues raised include the need for increasing public awareness of HEN; establishing national guidelines for nutrition; reporting of key performance indicators for HEN at a national or state level; and establishing centers within hospitals to manage HEN patients.

## 4. Discussion

This is the first Asia-Pacific region-specific HEN reimbursement and practice survey reported. Similar to other enteral nutrition surveys [11,22], healthcare regulations and nutritional guidelines are developed by government bodies and/or national societies. The presence of national nutrition societies is important for the advancement of clinical nutrition guidelines, as they contribute to publications of guidelines in most of the countries.

In comparison to Klek et al. [11], we were unable to find an association between countries’ income and HEN reimbursement. Nonetheless, we found that countries that spend a higher proportion of GDP on healthcare tend to reimburse HEN. However, this also indicates that there is a need to find a good balance between full reimbursement and co-payment to optimize the limited budget for healthcare, especially in countries with lower health expenditures. There may be a role for the private insurance sector in terms of HEN reimbursement, given that it only funds one-third of the countries with reimbursement. It is already known that adequate nutrition improves clinical outcomes in certain patient populations [28,29,30], and the provision of nutritional reimbursement is likely beneficial for the private insurance sector in the long-term.

Amongst the high-income countries, Brunei, Hong Kong, and Singapore do not have any reimbursement for HEN. For these countries, HEN is considered as food/consumables and therefore is not subsidized. In Singapore, some low-income patients/families may apply for a fund which can be used for HEN supplies. It is uncertain if there are similar provisions available in the other countries, as this survey did not set out to investigate the availability of special funding.

South Korea has implemented limited reimbursement for HEN in recent years. Baik [31] reported no HEN reimbursement in 2014, but in our current survey, it appears that partial reimbursement is now available. The reimbursement includes NST activities as a flat fee rate once weekly, although the authors reported that the amount reimbursed is too low and there is a lack of monitoring on the quality of care provided to patients.

The Japanese national health insurance provides HEN reimbursement across all healthcare areas and Japan has the second highest health expenditure in the region surveyed. Reimbursement for PEG insertion has increased from ¥64,000 yen per procedure in 2000 to ¥94,600 in 2002, which made it attractive to insert PEG in patients [17]. There were also anecdotal reports that some long-term care hospitals may decline admissions for patients unable to be fed orally, unless a PEG feeding tube is in situ [17]. This poses a dilemma for reimbursement, as we have to ensure that it is not exploited. Policy makers and clinical nutrition advocates have to ensure that fiscal responsibility and social justice are well balanced, as raised in a recent review by Martin and McGinnis [6].

Reimbursement in Australia varies greatly between jurisdictions and there appears to have been no changes made to the funding system since 2015 [16]. In New Zealand, the distribution of HEN reimbursement appears to be uniform across regions. However, it may be easier to implement nation-wide reimbursement in a country with a smaller population. Both Australia and New Zealand are amongst the highest spenders on healthcare in the region, and this also reflects in their financial support for HEN. Future challenges will include ensuring equitable distribution and access to HEN reimbursement in Australia, and maintaining fiscal prudence in New Zealand.

Amongst the upper-middle and lower-middle income countries, Indonesia is the only country that has some form of reimbursement for HEN, but it is still dependent on the patient’s state/city. Indonesia also has the lowest health expenditure in the 13 countries surveyed. The remaining countries of Cambodia, Malaysia, Myanmar, and the Philippines did not have any form of reimbursement for HEN, which is consistent with data on low and lower-middle income countries outside of Europe reported by Klek et al. [22].

Blenderized diet administered through feeding tubes remains a mainstay in these Southeast Asian countries, in particularly those of the lower-middle income tier. Additionally, the use of feeding tubes and the provision of commercial enteral feeds is not available in countries such as Cambodia and Myanmar, except in larger acute care hospitals in the capital cities. The use of blenderized diets via feeding tube has also been reported in other mid-income tier countries such as Thailand [32] and Brazil [33]. The use of blenderized diets needs to be properly managed, as there is increased risk of bacterial contamination and the increased viscosity causing occlusion of feeding tubes [34,35]. Although blenderized diets can be safely and effectively used [36], the lack of appropriate feeding tube adaptors and food preparation training in these countries will likely pose significant challenges.

There is a lack of NST and dietitians in lower-middle countries. The availability of NST is an important benefit for patient care, as shown in a recent systematic review [30], where nutrition support teams may be cost-effective for HEN support. This leads to an increased responsibility on doctors in these countries to manage the patients’ nutritional intake, in addition to providing medical care. More needs to be done in training new dietitians locally to assist the doctors in managing the nutritional status of patients for optimal care.

ESPEN Life Long Learning courses are well established in the Asia Pacific region, and are conducted in more than 70% of the countries where clinical nutrition training is available. The majority of the countries lack undergraduate courses in clinical nutrition, similar to recent international surveys [37,38], but postgraduate courses seem to be more readily available. In order to optimize clinical nutritional practice, it is necessary to include clinical nutrition training at the undergraduate level and continued training through to postgraduate. In the absence of established courses, the private sector (pharmaceutical companies) and local PEN societies need to step up and be more involved in the provision of clinical nutrition education.

As with all studies, there are limitations in our survey. There is likely a wide variability in practices and reimbursement between states/cities in the same country and respondents may be unaware of the differences, especially in the larger countries. In addition, some of the responders may have answered the survey based on their personal knowledge and not necessarily the actual national situation, leading to bias in the response. Almost every responder indicated that they are not aware of any governmental plans for HEN reimbursement, although information may have been released to only policy makers, who are not the target population for our survey.

The sample size was smaller than expected, as we were also unable to obtain participation from one-third of the countries. One of the reasons for non-response could be due to the way the survey was conducted by using an online survey form (Google Forms), and there are some countries where access to this site is banned. However, strengths of this study included that the distribution of countries based on their economic status covered a relatively wide range, and the survey results we obtained demonstrated the different national situations. Future surveys should include more countries and cities, as well as a wider range of survey respondents to ensure that the information collected relates to a more detailed nationwide situation.

## 5. Conclusions

This survey demonstrated the varied clinical and economic situation of HEN amongst the countries in the Asia Pacific region. There is a lack of reimbursement and clinical support for patients on HEN, and inadequate education opportunities for all the stakeholders, especially for the lower-middle income countries. Clinicians and local PEN societies need to lobby for more funding and convince governments of the clinical and cost effectiveness of HEN in order to provide the best care for patients.

## Figures and Tables

**Table 1:** Demographics of Countries Participating in Survey.

Country	Number of Hospitals	GDP (PPP)	GDP (US$)	Income Group	Health Expenditure (Public) (% GDP)	Body Responsible for Developing Healthcare Regulation	Body Responsible for Developing Nutritional Guidelines
Australia	101–1000	1,128,908	1,204,616	High	6.30%	State Health Services	State Health Services Various Organizations/Facilities
Brunei	<10	32,773	11,400	High	2.50%	Ministry of Health	Ministry of Health
Cambodia	101–1000	58,880	20,017	Lower-Middle	1.30%	Ministry of Health	None
Hong Kong SAR	10–100	430,169	320,912	High	5.70%	Department of Health	Department of Health
India	>1000	5,266,444	2,263,523	Lower-Middle	1.40%	Ministry of Health and Family Welfare	Various Clinical Societies
Indonesia	>1000	3,032,090	932,259	Lower-Middle	1.10%	Ministry of Health	Ministry of Health
Japan	>1000	5,266,444	4,939,384	High	8.60%	House of Representatives House of Councillors	Various Clinical Societies
Malaysia	101–1000	863,287	296,359	Upper-Middle	2.30%	Ministry of Health	Various Clinical Societies
Myanmar	>1000	305,301	67,430	Lower-Middle	1.00%	Ministry of Health	National Nutritional Centre Department of Public Health
New Zealand	10–100	183,291	185,017	High	9.10%	Government Health Boards	Ministry of Health
Singapore	10–100	492,631	296,966	High	2.10%	Ministry of Health	Health Promotion Board
Republic of Korea	>1000	1,832,073	1,411,246	High	4.00%	Ministry of Health and Welfare	Ministry of Health and Welfare
The Philippines	101–1000	806,539	304,905	Lower-Middle	1.60%	Department of Health	Department of Health

GDP: Gross Domestic Product; PPP: Purchasing Power Parity; SAR: Special Administrative Region.

**Table 2:** Healthcare Financing in Countries Participating in the Survey.

Country	State Health Insurance	Private Health Insurance	Financial Support for HEN (Full or Partial) Availability	Any Known Plans for Future HEN Funding
Australia	Yes	Yes	Yes	NA
Brunei	No	Yes	No	?
Cambodia	Yes	Yes	No	?
Hong Kong SAR	Yes	Yes	No	No
India	Yes	Yes	No	?
Indonesia	Yes	Yes	Yes	NA
Japan	Yes	No ^#^	Yes	NA
Malaysia	Yes	Yes	No	No
Myanmar	No	Yes	No	?
New Zealand	No	Yes	Yes	NA
Singapore	Yes	Yes	No	?
Republic of Korea	Yes	Yes	Yes	NA
The Philippines	Yes	Yes	No	?
Availability (Number, *n*)	9	12	5	0

HEN: home enteral nutrition; ?: Not Sure; NA: Not Applicable; ^#^: private insurance plays only a supplementary or complementary role.

**Table 3:** Countries where Financial Support is Available for Home Enteral Nutrition and/or Enteral Feeding in Acute Settings.

Country	Full or Partial Financial Support from State or Private Insurance	Coverage Area			
		Hospital	Long-Term Care Facilities	Palliative Care Facilities	Home
Australia ^#^	Partial/State	Some	Some	Some	Some
Indonesia ^#^	Partial/State Partial/Private	Some	Some	Some	Some
Japan	Partial/State	Yes	Yes	Yes	Yes
New Zealand	Full/State	Yes	Yes	Yes	Yes
Republic of Korea	Partial/State	Yes	Yes	Yes	Yes
Singapore *	Partial/State Partial/Private	Yes	No	No	No

**^#^** Availability of financial support varies between states/cities in the country; * Financial support is only available for certain patient paying classes in acute settings (government hospitals); Some: Available in some facilities.

**Table 4:** Home Enteral Nutrition Practices in Countries Participating in the Survey—Clinical Care.

Country	Profession Who Conducts Training for Patients/Family Members/Caregivers for Patients on HEN	Profession Who Prescribes Nutritional Care Plans	Availability of NST in			
			Hospital	Long-Term Care Facilities	Palliative Care Facilities	Home Care Support
Australia	Nurse (Hospital) Nurse (Private or Community) Dietitian	Dietitians	Some	No	No	No
Brunei	Nurse (Hospital) Nurse (Private or Community)	Dietitians	No	No	No	No
Cambodia	Nurse (Hospital) Doctor	Doctors	No	No	No	No
Hong Kong SAR	Nurse (Hospital) Nurse (Private or Community)	Dietitians	Some	No	No	No
India	Nurse (Hospital) Dietitian	Dietitians Nutritionists Doctors	Some	No	No	No
Indonesia	Nurse (Private or Community) Doctor Dietitian Nutritionist	Doctors	Some	Some	Some	Some
Japan	Nurse (Hospital) Doctor Dietitian	Dietitians Doctors	Some	Some	Some	Some
Malaysia	Nurse (Hospital) Caregiver training not provided	Dietitians	Some	No	No	No
Myanmar	Nurse (Private or Community) Doctor	Doctors	No	NA	No	No
New Zealand	Nurse (Hospital) Nurse (Private or Community) Dietitian External Vendors/Pharmaceutical Representatives	Dietitians	Some	No	No	No
Singapore	Nurse (Hospital) Nurse (Private or Community) Dietitian	Dietitians Doctors	Some	No	No	Some
Republic of Korea	Nurse (Hospital) Nurse (Private or Community) Doctor Dietitian	Dietitians Doctors	Some	Some	No	Some
The Philippines	Nurse (Hospital) Doctor Dietitian External Vendors/Pharmaceutical Representatives	Dietitians Doctors	Some	No	No	Some

HEN: Home Enteral Nutrition; NA: Not Applicable; **No**: Not Available; NST: Nutrition Support Team; **Some**: Available in some facilities.

**Table 5:** Clinical Nutrition Education in Countries Participating in the Survey. ESPEN: The European Society for Clinical Nutrition and Metabolism; PEN: Parenteral and Enteral Nutrition.

Country	Availability of Clinical Nutrition Training	Types of Clinical Nutrition Training Available						
		Undergraduate	Postgraduate	ESPEN Life Long Learning	Local—PEN Society organized	Local—Dietetic Association Organized	Local—Hospital Organized	Local—Pharmaceutical Organized
Australia	Yes	Yes	Yes	Yes	No	No	No	No
Brunei	No	No	No	No	No	No	No	No
Cambodia	No	No	No	No	No	No	No	No
Hong Kong SAR	Yes	No	Yes	Yes	Yes	Yes	No	No
India	Yes	No	No	No	No	Yes	Yes	Yes
Indonesia	Yes	No	Yes	Yes	Yes	Yes	No	No
Japan	Yes	Yes	Yes	Yes	Yes	Yes	Yes	Yes
Malaysia	Yes	No	No	Yes	Yes	Yes	Yes	No
Myanmar	Yes	No	Yes	No	No	No	Yes	No
New Zealand	Yes	No	Yes	No	No	No	Yes	No
Singapore	Yes	No	No	Yes	Yes	Yes	Yes	Yes
Republic of Korea	Yes	Yes	Yes	Yes	Yes	Yes	Yes	Yes
The Philippines	Yes	No	Yes	Yes	Yes	Yes	Yes	Yes
TOTAL	11	3	8	8	7	8	8	5

## Supplementary Materials

The following are available online at http://www.mdpi.com/2072-6643/10/2/214/s1.
