# Supplementary material for: A Survey of Home Enteral Nutrition Practices and Reimbursement in the Asia Pacific Region
Source: Nutrients. 2018 Feb 14;10(2):214. doi: 10.3390/nu10020214 (PMC5852790; doi:10.3390/nu10020214)
Supplement: Supplementary file 1 [file nutrients-10-00214-s001.pdf]

Supplementary Materials

Figure S1. Asia Pacific Home Enteral Nutrition Practice and Reimbursement Survey

SECTION 1. Introduction

The purpose of this study survey is to determine a) the presence of enteral nutrition usage in acute and chronic care settings, b) the type of enteral feeds used (commercial versus blenderized diets), c) the types of enteral access, d) the national and state reimbursement policies, and e) the presence of nutrition support teams for enteral nutrition support for the Asia Pacific region. The participation in this survey will greatly help us in understanding the policies and clinical practices in the Asia Pacific region, and guide us to future research studies required in this area.

SECTION 2. Demographics

a. Which country are you residing in?

☐ Australia
☐ Brunei
☐ Cambodia
☐ China
☐ Hong Kong
☐ India
☐ Indonesia
☐ Iran
☐ Japan
☐ Laos
☐ Macau
☐ Malaysia
☐ Myanmar
☐ New Zealand
☐ South Korea
☐ Singapore
☐ Sri Lanka
☐ Taiwan
☐ Thailand
☐ The Philippines
☐ Vietnam

b. Which State and/or City are you residing in? ______________________________

c. What is the population of your country? (Choose one answer)

☐ <1,000,000

☐ 1,000, 001 - 10,000,000

☐ 10,000,001 - 100,000,000

☐ >100,000,000

d. Number of hospitals (Private and Government) in your country (Choose one answer)

☐ <10

☐ 10 - 100

☐ 101-1000

☐ >1000

e. Health Expenditure as a Percentage of Gross Domestic Product (% GDP). Please refer to World Health Organization Global Health Expenditure database

http://data.worldbank.org/indicator/SH.XPD.PUBL.ZS ____

____

f. In your country, who is responsible for developing health regulations?

____

____

g. In your country, who is responsible for developing nutritional guidelines?

____

____

SECTION 3. Healthcare financing for Home Enteral Nutrition (HEN)

For the purpose of this survey, Home Enteral Nutrition (HEN) is defined as enteral tube feeding for patients who are unable to consume sufficient nutrients via the oral route.

a. What type of health insurance is available in your country? (Choose one answer)

☐ State/Government

☐ Private

☐ Both State and Private

b. Is the any financial support (full or partial) available for HEN in your country? NB: financial support in this question indicates funds available from either State/Government or Private Insurance. (Choose one answer)

☐ Yes

☐ No

c. Does financial support for HEN varies between the different States or Cities in your country? (Choose one answer)

☐ Yes

☐ No

SECTION 4. If financial support (full or partial) is NOT available for HEN in your country

If financial support for HEN varies between States or Cities, please answer the questions in this section based on the overall situation in the country. PLEASE SKIP THIS SECTION AND PROCEED TO SECTION 5 IF FINANCIAL SUPPORT IS AVAILABLE

a. Are there plans by the government for funding in the next couple of years? (Choose one answer)

☐ Yes

☐ No

b. Who pays for it? (more than one answer is possible)

☐ Patient and/or Family

☐ Hospital

☐ Hospital initiate charity funds

☐ Other charitable organizations

SECTION 5. If financial support (full or partial) is available for Home Enteral Nutrition (HEN) in your country

If financial support for HEN varies between States or Cities, please answer the questions in this section based on the situation in your State or City. PLEASE SKIP THIS SECTION AND ONLY COMPLETE SECTION 4 IF FINANCIAL SUPPORT IS NOT AVAILABLE.

a. Is financial support for HEN (more than one answer is possible)

☐ Fully paid by government

☐ Fully paid by private insurance

☐ Partially paid by government

☐ Partially paid by private insurance

b. Is financial support available for patients staying in HOSPITALS? (Choose one answer)

☐ Yes

☐ No

c. Is financial support available for patients staying in LONG-TERM CARE FACILITIES? (e.g. Nursing Homes, Homes for the Chronic Ill or Retirement Homes in your country)? (Choose one answer)

☐ Yes

☐ No

☐ No Long-Term Care facilities in the country

d. Is financial support available for patients staying in PALLIATIVE CARE FACILITIES? (Choose one answer)

☐ Yes

☐ No

☐ No Palliative Care facilities in the country

e. Is financial support available for patients staying in OWN HOMES? (Choose one answer)

☐ Yes

☐ No

f. Is the source of financial support for inpatient different from outpatient? (Choose one answer)

☐ Yes

☐ No

SECTION 6. Home Enteral Nutrition Practices

Please answer the questions in this section based on the situation nationally.

a. Is HEN available readily in your country? (I.e. are feeding tubes used for patients who cannot eat orally in home /long-term care settings?) (Choose one answer)

☐ Yes

☐ No

b. Who conducts training for patients/ family members/ caregivers for patients who needs to be discharged from the hospital with HEN? (more than one answer is possible)

☐ Nurse (Hospital)

☐ Nurse (Private or Community)

☐ Doctor

☐ Dietitian

☐ Nutritionist

☐ External Vendors/ Pharmaceutical Representatives

☐ Caregiver training not provided

c. For patients on HEN at HOME, which types of feeds are used? (Choose one answer)

☐ Mainly Commercial Feeds/Supplements

☐ Mainly Blenderized Diets

☐ About equal proportions of Commercial Feeds and Blenderized Diets

☐ Patients do not use commercial feeds/supplements at home

d. For patients on HEN at HOME who uses Commercial Feeds/Supplements, do they use mainly? (Choose one answer)

☐ Liquid ready-to-use formula

☐ Powdered feeds which is reconstituted at home

☐ Both Liquid and/or Powdered

☐ Patients do not use commercial feeds/supplements at home

e. For patients on HEN in LONG-TERM CARE SETTINGS, which types of feeds are used? (Choose one answer)

☐ Liquid ready-to-use formula

☐ Powdered feeds which is reconstituted at home

☐ Both Liquid and/or Powdered

☐ Patients do not use commercial feeds/supplements in long term care settings

f. For patients on HEN in LONG-TERM CARE SETTINGS who uses Commercial Feeds/Supplements, do they use mainly (Choose one answer)

☐ Liquid ready-to-use formula

☐ Powdered feeds which is reconstituted at home

☐ Both Liquid and/or Powdered

☐ Patients do not use commercial feeds/supplements in long term care settings

SECTION 7. Statistics for HEN

Please answer the questions in this section based on the situation nationally.

Are there NATIONAL statistics available for:

A) Incidence of HEN (Cases per 1 million inhabitants per year) or

B) Prevalence of HEN (Cases per 1 million inhabitants) or

C) No data available.

Please write down all figures if available.

a. Gastrostomy Tube

b. Nasoenteric Tube

SECTION 8. Nutritional Support Teams

Nutritional Support Team is a multi-disciplinary team of Doctors, Dietitians, Nurses and Pharmacists. (NB: A team is considered present if there are at least 3 different healthcare professionals.)

Please answer the questions in this section based on the situation nationally.

a. Who prescribes nutritional care plans in your country's hospital? (more than one answer is possible)

☐ Dietitian
☐ Nutritionist
☐ Nurse
☐ Doctor
☐ Other Healthcare Professionals

b. Are there Nutrition Support teams in Hospitals? (Choose one answer)

☐ Yes, in all hospitals
☐ Yes, in some hospitals
☐ No Nutrition Support Teams

c. Are there Nutrition Support teams in Chronic Care Facilities? (Choose one answer)

☐ Yes, in all facilities
☐ Yes, in some facilities
☐ No Nutrition Support Teams
☐ No Chronic Care Facilities in the country

d. Are there Nutrition Support teams in Palliative Care Facilities? (Choose one answer)

☐ Yes, in all facilities
☐ Yes, in some facilities
☐ No Nutrition Support Teams
☐ No Palliative Care Facilities in the country

e. Are there Nutrition Support teams in Home Care Support? (Choose one answer)

☐ Yes, in all facilities

☐ Yes, in some facilities
☐ No Nutrition Support Teams
☐ No Home Care Support in the country

SECTION 9. Clinical Nutrition Training

Please answer the questions in this section based on the situation nationally.

a. Is there any training in clinical nutrition for healthcare professionals in your country? (Choose one answer)
☐ Yes
☐ No

b. What type of clinical nutritional training is available? (more than one answer is possible)
☐ Undergraduate
☐ Postgraduate
☐ ESPEN LLL (lifelong learning)
☐ Local – PEN Society organized
☐ Local – Hospital organized
☐ Local – Pharmaceutical organized
☐ Local - Dietetic Association organized
☐ None Available

SECTION 10. Further Support

Please answer the questions in this section based on the situation nationally.

a. In your opinion, what is urgently required for HEN support in your country? This can be in terms of training or funding resources, public awareness programs, clinical knowledge and training of staff/family/patient, Governmental /Non-Governmental Organization/ Private Company support

Table S1. Types of Feeds Used in the Countries Surveyed

Country	Is HEN available readily	Type of Feeds Used for Patients on HEN at HOME	Type of Commercial Feeds Used for Patients on HEN at HOME	Type of Feeds Used for patients on HEN in LONG-TERM CARE	Type of Commercial Feeds Used for Patients on HEN in LONG-TERM CARE
Australia	~~+~~Yes	Mainly Commercial	Liquid ready-to-use	Mainly Commercial	Liquid ready-to-use
Brunei	Yes~~+~~	Mainly Commercial	Powdered feeds	Mainly Commercial	Powdered feeds
Cambodia	Yes~~+~~	Mainly Blenderized	Both Liquid and/or Powdered	Mainly Blenderized	Both Liquid and/or Powdered
Hong Kong SAR	Yes~~+~~	Mainly Commercial	Liquid ready-to-use	Mainly Commercial	Liquid ready-to-use
India	Yes~~+~~	Similar proportions of Commercial and Blenderized	Powdered feeds	Similar proportions of Commercial and Blenderized	Powdered feeds
Indonesia	Yes~~+~~	Similar proportions of Commercial and Blenderized	Powdered feeds	Mainly Blenderized	Both Liquid and/or Powdered
Japan	Yes~~+~~	Mainly Commercial	Liquid ready-to-use	Mainly Commercial	Liquid ready-to-use
Malaysia	Yes~~+~~	Mainly Commercial	Powdered feeds	Mainly Commercial	Powdered feeds
Myanmar	Yes~~+~~	Similar proportions of Commercial and Blenderized	Both Liquid and/or Powdered	Similar proportions of Commercial and Blenderized	Both Liquid and/or Powdered
New Zealand	Yes~~+~~	Mainly Commercial	Liquid ready-to-use	Mainly Commercial	Liquid ready-to-use
Singapore	Yes~~+~~	Mainly Commercial	Both Liquid and/or Powdered	Mainly Commercial	Both Liquid and/or Powdered
Republic of Korea	Yes~~+~~	Mainly Commercial	Liquid ready-to-use	Mainly Commercial	Liquid ready-to-use
The Philippines	~~-~~No	Mainly Blenderized	Both Liquid and/or Powdered	Mainly Blenderized	Both Liquid and/or Powdered

~~+: Yes; -: No~~
